# Circadian influences on glycemic control in 24-hr fasts initiated at different mealtimes

**DOI:** 10.3389/fnut.2026.1779113

**Published:** 2026-04-15

**Authors:** Elizabeth Z. Gipson, G. Chretienne Kang, Katelynn E. Hales, Dallin J. Kemp, Dane T. Jensen, Lance E. Davidson, Larry A. Tucker, Bruce W. Bailey

**Affiliations:** Lifestyle Medicine Lab, Department of Exercise Science, Brigham Young University, Provo, UT, United States

**Keywords:** fasting, glucagon, glucose tolerance, glycemic control, insulin, ketosis, metabolic health, OGTT

## Abstract

**Introduction:**

This study investigated whether the timing of a 24-h fast influences indicators of glycemic control throughout the duration of the fast or upon refeeding.

**Methods:**

Twenty-four adults who have overweight or obesity (13 female, 11 male) completed this randomized crossover study involving three 24-h fasts initiated at either 8:00 am, 1:00 pm, or 6:00 pm followed by a 75 g oral glucose tolerance test (OGTT) which lasted 2 h. Continuous glucose monitors (CGMs) were employed to track interstitial glucose throughout each fast and OGTT. Plasma samples for insulin and glucagon were collected at the beginning and end of each fast, and 30 min post-OGTT. Capillary beta-hydroxybutyrate (BHB) was only measured at the end of the fasting.

**Results:**

The 24-h fast initiated in the afternoon had lower post-prandial glucose area under the curve (AUC) following a standardized meal and lower mean 24-h glucose levels than the morning or evening fasts (*p* < 0.0001) which did not differ. Glucose AUC above baseline at the conclusion of a fast was optimal after the morning compared to the afternoon or evening fast (*p* < 0.0001), though BHB was above the ketosis threshold (≥0.5 mmol/L) at the end of the afternoon and evening fasts but not the morning fast. Insulin and glucagon did not differ at any point (*p* > 0.05).

**Discussion:**

The timing of a 24-h fast alters glycemic control and metabolic switching. The afternoon fast optimized glycemic outcomes and ketosis, and these effects appear independent of insulin and glucagon. Aligning fasting protocols with circadian rhythms by initiating a 24-h fast in the afternoon may improve metabolic outcomes related to glycemic control during and immediately after the fast.

## 1 Introduction

Effective glycemic control is a critical aspect of metabolic health. Metabolically healthy individuals exhibit lower postprandial blood glucose peaks and faster blood glucose clearance than those who are metabolically unhealthy (1). They also have greater insulin sensitivity, meaning their bodies are more responsive to insulin’s signal to uptake blood glucose into cells (2). Proper glycemic control can reduce the risk and severity of cardiometabolic diseases (3) and certain cancers (4), as lower glucose peaks and higher insulin sensitivity mitigate the negative impact that hyperglycemia can have on the body. Additionally, lower insulin levels reduce the stimulus for energy storage in the body to instead favor energy expenditure, reducing the risk of obesity and obesity-related chronic diseases (5).

Fasting is one strategy used to improve glycemic control. As hepatic glycogen stores become depleted (6) and insulin secretion decreases, the body utilizes more fat-derived energy substrates, such as ketones, to meet its energy needs (7, 8). Fasting also enhances the body’s sensitivity to insulin-stimulated glucose uptake and the responsiveness of pancreatic beta cells (9). Together, these factors contribute to lower post-prandial glucose peaks and more rapid glucose clearance upon refeeding.

The timing of both food intake and fasting windows influence the impact of circadian rhythms on glycemic control (10). Post-prandial concentrations of insulin increase more rapidly in the morning (11–13). Likewise, beta cell responsiveness and insulin sensitivity, and therefore glucose tolerance, are higher in the morning compared to the evening (8, 11–14).

Given these circadian variations, consuming calories in the morning appears to be most beneficial for glycemic control, as the body’s response to glucose ingestion is more efficient (10). Conversely, consuming calories in the evening is less favorable due to a slower glycemic response. Extending the amount of time spent fasting in the evening may be more advantageous for metabolic health since overnight glucose levels tend to be lower when food intake occurs earlier in the day (10). By aligning eating patterns with the body’s circadian rhythm for glycemic control, metabolic health can be enhanced.

Although several studies have evaluated the role of circadian rhythms in glucose tolerance following an OGTT in the morning, afternoon, and evening, the fasting durations in these protocols were relatively short. Extending the fasting period prior to an OGTT to 24-h eliminates post-prandial influences and provides a clearer assessment of circadian impacts on glucose tolerance. Despite growing interest in intermittent fasting protocols, there is limited research exploring the impact of circadian rhythms on glucose regulation, meaning regulation of normoglycemia, during and glucose tolerance after a 24-h fasting period. Investigating how the timing of a 24-h fast impacts these aspects of glycemic control can offer valuable insights for those seeking to optimize fasting strategies for metabolic benefits.

The purpose of this study was to determine whether the 24-h glycemic response differs between the time of day the fast was initiated. The study also assessed how 24-h fasting start times influence glucose tolerance, hormonal responses of insulin and glucagon, and beta-hydroxybutyrate (BHB) concentrations at the conclusion of each fast. We hypothesized that glucose regulation during the fast and glucose tolerance after the fast would be more favorable for the morning fast, followed by the afternoon and then the evening fasts. We also hypothesized that there would be a greater insulin and incretin response to an OGTT after the morning fast, followed by the afternoon and then evening fasts.

## 2 Materials and methods

### 2.1 Design

This study used a randomized crossover design with three counterbalanced treatment conditions. Each condition required participants to complete a 24-h water-only fasting period followed by an OGTT. The fasting periods were scheduled to begin and end at three distinct times: 8 am, 1 pm, and 6 pm. The effects of fast timing on ketones and glycemic hormones, including insulin and glucagon was evaluated. The study protocol received approval from the Institutional Review Board at Brigham Young University (IRB2023-224).

Participants completed all three fasting conditions, with a 5-days washout period between each. The order of fasting conditions was randomly assigned to participants using (15), with participant numbers assigned chronologically based on the order of enrollment. Before each fasting condition, participants were screened for contraindications to ensure continued eligibility. The following outcome variables were assessed before starting any fast: body mass index (BMI), body fat percentage (BF%), visceral adipose tissue (g), venous plasma concentrations of the aforementioned glycemic hormones, and capillary BHB.

### 2.2 Participants

A total of 25 participants (13 males and 12 females) were recruited through flyers, advertisements, social media platforms, word-of-mouth, and informal referrals. Eligibility criteria included individuals aged 18 years or older with a reported BMI greater than 27 who had maintained a stable body weight (±5% of body weight) over the preceding 3 months (16). No maximum age was established for study participants. Table 1 reports the demographic and anthropometric characteristics of those participating in the study, including the 24 individuals who completed the study and the one individual who dropped after completing one condition. Data from each completed fast were analyzed in this study.

**TABLE 1:** Demographic and anthropometric characteristics of participants.

	Female (*n* = 13)		Male (*n* = 12)		Total (*n* = 25)	
Characteristic	Mean	SD	Mean	SD	Mean	SD
Age (years)	32.23	14.04	34.83	12.51	33.48	13.12
BMI (kg/m^2^)	31.09	5.23	32.37	3.68	31.70	4.51
BF%	42.65	6.17	34.94	8.17	38.95	8.07
Visceral adipose tissue (g)	812.54	531.86	1771.08	973.32	1272.64	902.51
Ethnicity	*n*	%	*n*	%	*n*	%
Caucasian	10	76.9	11	91.67	21	84
Hispanic	1	7.7	1	8.33	2	8
Black	1	7.7	–	–	1	4
Asian	1	7.7	–	–	1	4

Individuals meeting any of the following exclusion criteria were ineligible to participate in the study:

- Chronic or metabolic disease diagnosis (e.g., cardiovascular, renal, hepatic conditions, or cancer).
- Eating disorder diagnosis.
- Use of medications that affect appetite, metabolism, or neurological function (e.g., metformin, insulin, amphetamine-based ADHD medications, antidepressants, or anti-anxiety medications) (17).
- Regular caffeine consumption of 60 mg or more per day (18).
- Known food allergies.
- Pregnancy or lactation.
- Current adherence to calorie-restricted, low-carbohydrate, or ketogenic diets.

Engaging in more than 225 min of exercise per week.

Non-habitual breakfast consumption.

Self-reported habitual bedtimes after 12:30 am or waketimes after 8:30 am.

Prospective participants received an email containing a hyperlink to an online survey for the initial screening process. The survey was designed to confirm eligibility based on the exclusion criteria. Qualified participants were invited to participate in the study and were instructed to abstain from caffeine, stimulants, and vigorous physical activity for 24 h prior to and throughout each testing period. Adherence to pre-test protocols was verified at the start of each fasting session. Non-compliance resulted in the participant being rescheduled.

### 2.3 Orientation

Participants provided informed consent prior to participation in any aspect of the study. All testing sessions were conducted at the Human Performance Research Laboratory at Brigham Young University. During the initial orientation, participants were briefed on the primary objectives of the study and familiarized with the testing procedures. They were instructed to maintain their regular daily routines, avoid exercise or strenuous activities, and adhere to their usual sleep patterns. Participants also received text message reminders about their appointments the day before their scheduled visit.

### 2.4 Treatment sessions

Before each fasting session, participants were instructed to follow their usual dietary habits but to consume their final meal at least 4 h before the start of the fast. This timing was based on evidence that postprandial glucose typically returns to baseline within approximately 2 h, while insulin concentrations generally return to baseline within about 4 h after a meal.

Before each fasting session, participants were instructed to follow their usual dietary habits but to consume their final meal at least 4 h before the start of the fast. This timing was based on evidence that postprandial glucose typically returns to baseline within approximately 2 h, while insulin concentrations generally return to baseline within about 4 h after a meal (5). The purpose of this instruction was to minimize the likelihood that participants would intentionally or unintentionally preload the fast and to promote more comparable pre-fast conditions across sessions. Given these precautions, dietary intake was not assessed prior to the fasting periods.

Participants reported to the Human Performance Laboratory 1 h before their first assigned fasting condition. The fasting start time was determined by random assignment, with participants beginning their fast at either 8 am, 1 pm, or 6 pm. During this initial visit, informed consent was obtained, a dual-energy X-ray absorptiometry (DXA) scan was performed, and participants were given a CGM and an accelerometer approximately 30 min before the fast start time. At the designated fasting start time, a baseline venous blood sample was collected, and participants consumed a standardized meal within 30 min, initiating the 24-h fasting period.

Participants completed each 24-h water-only fast at home. For each condition, they were instructed to maintain typical daily activities and to keep a consistent sleep schedule. Medications that did not alter metabolism were permitted if consumed at the same time each day. Participant compliance to fasting protocols was verified through CGM monitoring. At the conclusion of each 24-h fast, a second venous blood sample was collected.

At the conclusion of the fast, participants were given a 75 g OGTT and remained in the lab for 2 h. Another blood draw was taken 30 min after consuming the OGTT. The participants remained sedentary in the lab for the duration of the visit, monitored by research assistants.

### 2.5 Measurements

#### 2.5.1 Anthropometric measurements

The day the first fast began, participants’ height and body weight were measured. Height was recorded using a stadiometer with an accuracy of ±0.1 cm (Seca, Hamburg, Germany), while weight was measured using a digital scale with an accuracy of ±0.1 kg (Seca, Hamburg, Germany). Measurements were taken with participants dressed in light clothing. Body mass index (BMI) was calculated as weight (kg) divided by height squared (m^2^). Percent body fat was assessed using a GE iDXA system (GE, Fairfield, CT) (19–21). The scanner was calibrated prior to each use with a manufacturer-provided calibration block, and scans were analyzed using Encore software version 17.

#### 2.5.2 Venipuncture

Blood samples were collected immediately before the start of the fast (*t* = 0 hrs) and at the end of the fast (*t* = 24 hrs). Each sample was collected into an EDTA Vacutainer tube, centrifuged at 1500 × *g* for 15 min to separate plasma, then pipetted into cryovials. A protease inhibitor (Halt™ Protease Inhibitor Cocktail 100×, Thermo Fisher Scientific, Inc.) was added to the plasma samples. The cryovials were subsequently stored at −80 °C for future analysis.

Concentrations of insulin and glucagon were determined using standard curves created from diluted standard fluorescence values obtained from running Human Metabolic Hormone Magnetic Bead Panel multiplex kits (Cat. #HMHEMAG-34K) through a MAGPIX™ Multiplex Reader (Luminex Corporation, Austin, Texas, USA). Concentrations for each plasma sample value were interpolated for each hormone.

#### 2.5.3 Continuous glucose monitoring

To assess glucose levels throughout the fasting periods, the Freestyle Libre Pro (Abbott Laboratories), which collects data for a 14-days period, was used. The continuous glucose monitor (CGM) was inserted on the back of the upper arm to ensure sensor accuracy (22). The CGM measures interstitial glucose concentrations, which reflects plasma glucose concentrations (23). Interstitial glucose concentrations were measured every 15 min. The data were downloaded using a wireless scanner that uploaded the date to online cloud storage for subsequent analysis. The accuracy and reliability of the Freestyle Libre Pro has been validated compared to other glucose measurements including the Yellow Spring Instrument standard (24–26).

### 2.6 Oral glucose tolerance test

The OGTT was composed of 300 mL of water containing 75 g of glucose in the form of dextrose (27, 28). At the conclusion of each fasting condition, participants drank the OGTT within 5 min then remained seated in the laboratory for the next 2 h (29). Participants were asked to refrain from eating throughout the duration of the test. Variations in interstitial glucose concentrations over the 2-h OGTT along with peak glucose concentrations and area under the curve (AUC) were measured using the participant’s CGM. This approach has been previously validated as a method to measure glucose concentrations during an OGTT (27, 30). In this study, participants used the same CGM for the duration of the study.

### 2.7 Capillary ketone assessment

Ketones were measured using the Precision Xtra portable ketone meter (Abbott Laboratories, Abington, UK). An electrochemical chip was inserted into the center and applied to the capillary blood sample following a finger prick to quantify BHB concentrations at the conclusion of each fast (time = 24 hrs). Bryne et al. demonstrated that the Precision Xtra portable ketone meter is accurate for BHB concentrations up to 6 mmol/L when comparing capillary BHB to venous blood reference samples. The mean difference between sensor and reference values was +0.02 mmol/L, with a range from −0.6 to +0.6 mmol/L. The reproducibility standard deviation was reported as 0.13 mmol/L (31).

### 2.8 Standardized meals

Prior to each fasting session, participants were provided with a standardized meal designed to provide 25% of the participant’s calculated daily energy requirements. Such requirements were estimated using validated equations via the online NIH body weight planner (32, 33) that incorporate height (cm), weight (kg), age (years), and sex to predict basal metabolic rate (BMR). To calculate total daily energy needs, an activity factor of 1.4 was applied (BMR × 1.4 × 0.25) (34). Macronutrient composition was standardized at 60% carbohydrates, 25% fat, and 15% protein, and consisted of various combinations of pre-packaged peanut butter and jelly sandwiches, crackers, almonds, string cheese, and beef jerky. Meals were identical across sessions, and participants were required to consume the entire meal. Adherence was monitored by researchers in the laboratory.

### 2.9 Statistical analysis

A sample size of 24 participants was determined to achieve 90% power to detect an effect size of 0.61 (35) at α = 0.05, with recruitment continuing until all participants completed the study. Mixed models were implemented using PROC MIXED in SAS (version 9.4; SAS Institute, Cary, NC) to analyze the effects of experimental conditions (24-h fast starting at 8 am, 1 pm, or 6 pm) and time on glucose and biomarker outcomes.

Glucose levels were measured every 15 min (97 observations over 24 h), while insulin and glucagon were measured at 0, 24, and 24.5 h. BHB was only measured at 24 h. A mixed model assessed the main and interactive effects of condition, time, and the interaction between condition and time, with fast order as a covariate and participant as a random effect. A compound symmetry covariance structure modeled within-subject correlations, with REML estimation and Kenward-Roger degrees of freedom. Separate models using the same analysis were fitted for BHB, insulin and glucagon.

To evaluate glucose area under the curve (AUC), calculated using the trapezoidal rule, mixed models included condition and order as fixed effects and participant as a random effect, with an unstructured covariance structure. REML estimation and Kenward-Roger degrees of freedom were applied. Least squares means for condition effects were compared with Tukey-adjusted pairwise tests. Condition order was utilized as a covariate for all analyses, with no significant impact on any results.

Results were reported as standard estimates ± standard error of the estimates in the paper, and data are presented as means ± standard deviations in each table, with significant effects (*p* ≤ 0.05) further analyzed using Tukey-adjusted *post hoc* comparisons. *F*-values indicated main and interactive effects, while *t*-values reflected pairwise comparisons. All analyses were conducted in SAS 9.4.

## 3 Results

**FIGURE 1:**
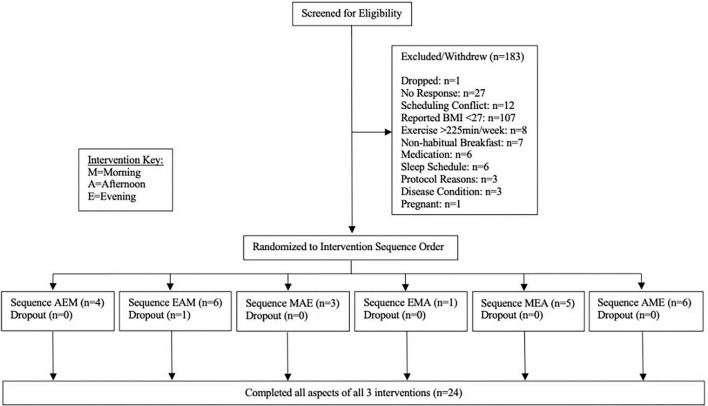
Participant flow chart.

A total of 182 prospective participants were assessed for eligibility (see Figure 1). The majority were excluded based on low BMI, scheduling conflicts, or failure to respond after completing the survey. Ultimately, 25 qualifying individuals began the study and 24 completed all aspects of the study. One individual withdrew from the study after completing a single condition due to bruising from blood draws. Data from that completed condition were reported. Participants consumed 639.3 ± 94.1 calories in each standardized meal.

### 3.1 Glucose

**FIGURE 2:**
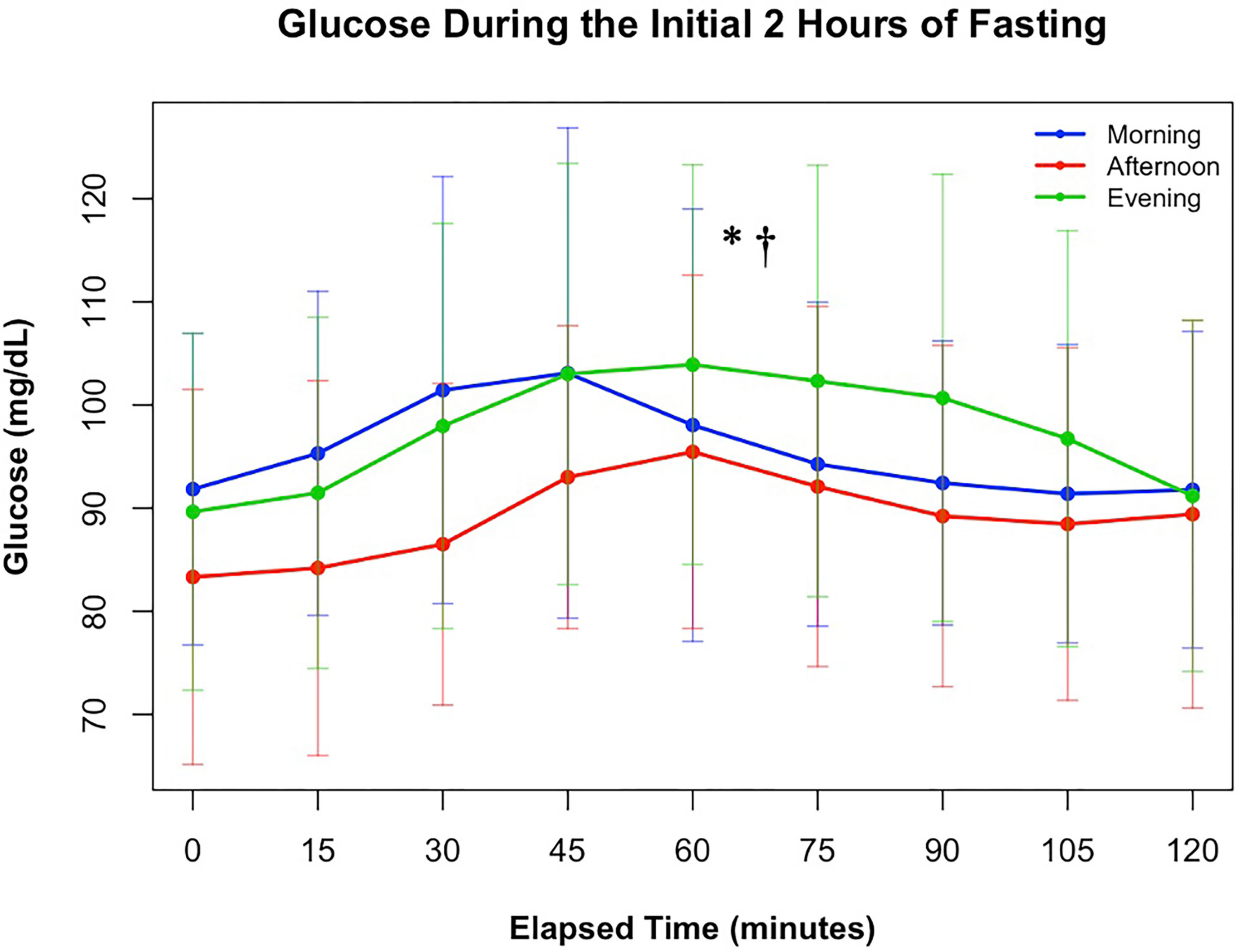
Changes in glucose during the first 2 h of fasting following a standardized meal based on time of fast. * indicates a pairwise comparison with a significant difference between the morning and afternoon fasts. † indicates a pairwise comparison with a significant difference between the evening and afternoon fasts.

The mean glucose concentration at the beginning of each fast did not differ between conditions [*F*(2, 21.7) = 1.02, *p* = 0.3779]. A standardized meal was provided to initiate the fast and the 2-h glucose response to this meal during each was analyzed (see Figure 2). Portions of data were missing in 8 conditions due to random CGM activation delays. While there was not a condition by time interaction during the first 2 h of each fast [*F*(16, 550) = 1.24, *p* = 0.2322], there was a main effect of condition [*F*(2, 556) = 12.11, *p* < 0.0001]. Glucose was lower in the afternoon than the morning [t(553) = −4.01, *p* < 0.0001] and evening [t(558) = −4.48, *p* < 0.0001]. 2-h glucose did not differ between the morning and evening conditions [t(557) = 0.61, *p* = 0.5404].

**FIGURE 3:**
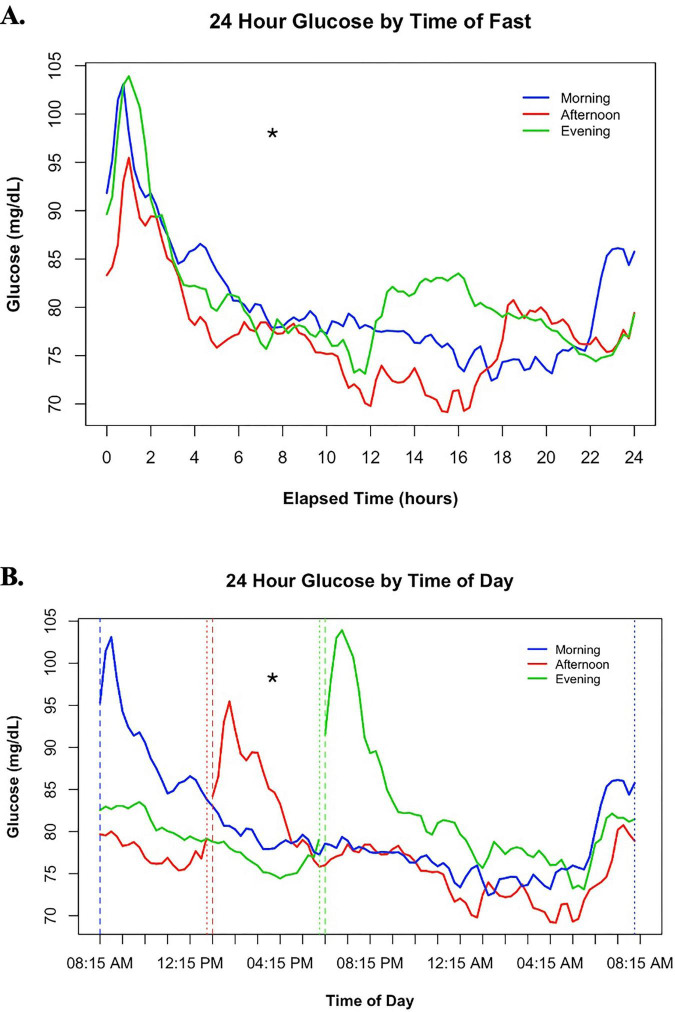
**(A)** Shows changes in glucose over each 24-h fasting period based on the relative time of fast. **(B)** Shows changes in glucose from 8:15 am–8 am in the morning condition, 1:15 pm–1 pm in the afternoon condition, and 6:15 pm–6 pm in the evening condition based on the actual clock time of day. Baseline values were excluded to allow comparison of glucose patterns at similar times of day. Dashed lines indicate glucose values closest to fast initiation, and dotted lines indicate the end of fasting for their respective conditions. * indicates a significant condition by time interaction with lower glucose in the afternoon fast than the morning or evening fasts for the 24-hr period.

A separate analysis comparing glucose levels over each 24-h period matched by time of day was also conducted. Portions of data extending slightly beyond 2 h were missing in 5 conditions, due to random CGM activation delays. In this analysis, a significant condition-by-time interaction [*F*(190, 6409) = 6.44, *p* < 0.0001] and main effect of condition [*F*(2, 6411) = 76.46, *p* < 0.0001] were observed. Over the course of the whole fast mean glucose during the afternoon fast was 3.3 ± 0.3 mg/dL lower than the morning fast [t(6409) = −10.53, *p* < 0.0001] and 3.4 ± 0.3 mg/dL lower than the evening fast [t(6413) = −10.81, *p* < 0.0001], with no difference between morning and evening [t(6413) = 0.52, *p* = 0.6027) (see Figure 3).

We also divided the 24-h fast into four time periods: Morning (8:15 am–1:00 pm), Afternoon (1:15 pm–6:00 pm), Evening (6:15 pm–11:00 pm) and Nighttime (11:15 pm–6:00 am). Glucose during the fast initiated during the morning, afternoon, or evening time periods was expected to peak from consuming the standardized meal. Therefore, the purpose of this division was to compare daytime glycemic responses in the two conditions without an immediate post-prandial influence during a particular window and to assess glucose responses across all conditions in the nighttime. The following are the results for the main effect of condition during each of these time periods:

Morning [*F*(2, 1377) = 160.22, *p* < 0.0001]: glucose was higher for the morning fast than the afternoon [12.6 ± 0.7 mg/dL; t(1375) = 17.37, *p* < 0.0001] or evening fast [9.3 ± 0.7 mg/dL; t(1378) = −12.55, *p* < 0.0001]. Glucose was also higher for the evening fast than the afternoon fast [3.4 ± 0.7 mg/dL; t(1378) = 4.62, *p* < 0.0001] (see Figure 3B).

Afternoon [*F*(2, 1343) = 67.39, *p* < 0.001]: glucose was higher for the afternoon fast than the morning [5.0 ± 0.7 mg/dL; t(1341) = 7.60, *p* < 0.0001] or evening fast [8.0 ± 0.7 mg/dL; t(1349) = 11.49, *p* < 0.0001]. Glucose was also higher in the morning fast than the evening fast [3.1 ± 0.7 mg/dL; t(1344) = −4.70, *p* < 0.0001] (see Figure 3B).

Evening [*F*(2, 1331) = 182.47, *p* < 0.0001]: glucose was higher for the evening fast than the morning [11.1 ± 0.7 mg/dL; t(1332) = 15.12, *p* < 0.0001] or afternoon fast [12.0 ± 0.7 mg/dL; t(1332) = −17.70, *p* < 0.0001], which were not different [0.9 ± 0.7 mg/dL; t(1328) = −1.27, *p* = 0.2036] (see Figure 3B).

Nighttime [*F*(2, 1913) = 67.37, *p* < 0.0001]: glucose was higher for the evening fast than the morning [3.0 ± 0.6 mg/dL; t(1914) = 4.77, *p* < 0.0001] or afternoon fast [5.9 ± 0.6 mg/dL; t(1914) = −11.61, *p* < 0.0001]. Glucose was also higher in the morning fast than the afternoon fast [2.9 ± 0.6 mg/dL; t(1911) = 4.73, *p* < 0.0001] (see Figure 3B).

### 3.2 OGTT AUC

**FIGURE 4:**
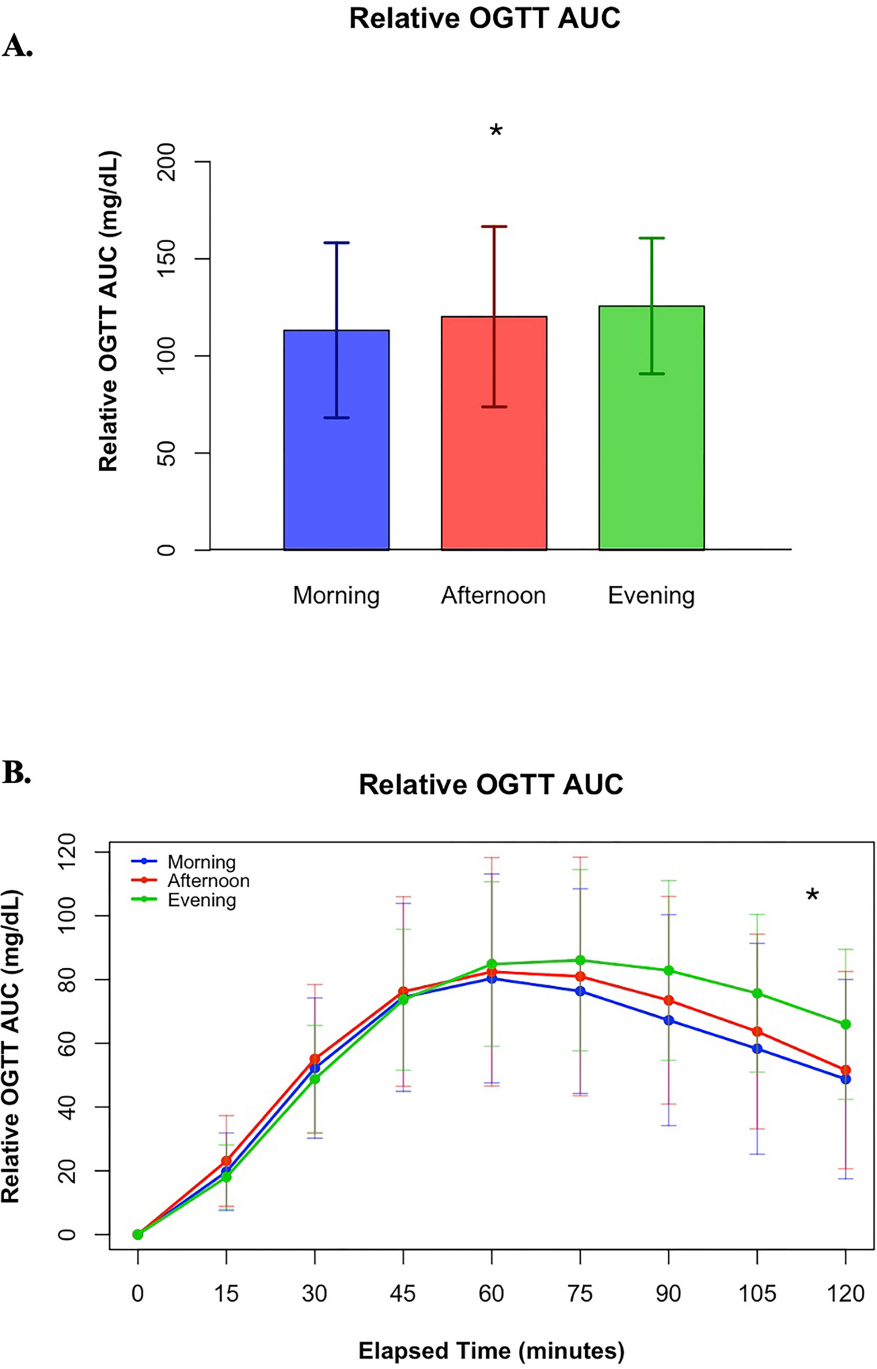
**(A)** Total OGTT AUC above baseline glucose levels and **(B)** OGTT AUC above baseline levels over time. * indicates a pairwise comparison with a significant difference between the morning and evening fasts.

A 75 g oral glucose tolerance test (OGTT) was performed at the end of each 24-h fast to evaluate glucose tolerance. Glucose levels were measured every 15 min for 2 h using continuous glucose monitors (CGMs), and the area under the curve (AUC) was calculated using the trapezoidal rule. The analysis included 73 fasting conditions; however, CGM data were missing for 18 conditions due to premature removal of monitors before complete data consolidation. Despite the 24-h fasting lead-in for each condition, glucose levels were higher in the morning fast prior to the OGTT compared to the afternoon or evening fasts [t(6409) = 2.11, *p* = 0.0346; t(6409) = 2.21, *p* = 0.0274, respectively], which were similar to each other [t(6409) = 0.09, *p* = 0.9264]. To account for this, we evaluated the relative rise in glucose above baseline. When evaluating the relative glucose response over the 2 h following the OGTT, there was a main effect of condition [*F*(2, 28.8) = 3.62, *p* = 0.0395] for glucose AUC. Relative glucose AUC was similar in the morning and afternoon conditions [t(30.2) = 1.98, *p* = 0.0568], but lower in the morning than the evening conditions [t(29.8) = 2.65, *p* = 0.0128]. Relative glucose AUC was similar between the afternoon and evening conditions as well [t(27) = −0.96, *p* = 0.3481] (see Figure 4). Glucose AUC above baseline was 96.9 ± 11.6 mg/dL in the morning, 114.7 ± 11.1 mg/dL in the afternoon, and 128.5 ± 8.3 mg/dL in the evening.

### 3.3 Glycemic hormones

Hormone concentrations are reported in Table 2. Of these, one value from 30 min post OGTT for glucagon, and three values from baseline and from 24 h for insulin were missing due to inability to extrapolate data from the plasma samples. We also were unable to complete a blood draw at 24-h for one individual in the morning condition. There was no significant condition by time interaction for insulin [*F*(4, 179) = 0.51, *p* = 0.7309] or glucagon [*F*(4, 184) = 1.35, *p* = 0.2539]; however, there was a main effect of time for insulin [*F*(2, 179) = 103.57, *p* < 0.0001] and glucagon [*F*(2, 184) = 4.20, *p* = 0.0165]. There was no difference in insulin or glucagon from pre- to post-fasting [t(179) = 1.21, *p* = 0.2263; t(184) = −1.14, *p* = 0.4920, respectively], but 30 min after consuming the OGTT, insulin rose above pre- and post-fasting levels [t(179) = −12.96, *p* < 0.0001; t(179) = −11.77, *p* < 0.0001] and glucagon rose above pre-fasting levels [t(184) = −2.88, *p* = 0.0045] but not post-fasting levels [t(184) = −1.73, *p* = 0.0847]. There was no main effect of condition for either insulin [*F*(2, 180) = 0.18, *p* = 0.8365] or glucagon [*F*(2, 185) = 0.51, *p* = 0.5992].

**TABLE 2:** Concentrations of insulin and glucagon (pg/mL) at various time points in each condition.

Hormone	Condition	0 h	24 h	24.5 h	*F*-value	*p*-value
Insulin (pg/mL)	Morning	2174.4 ± 2546.8	1886.2 ± 2449.3	6993.6 ± 4688.7^a,b^	0.51	0.7309
	Afternoon	2036.4 ± 2722.6	1690.7 ± 2491.8	6418.3 ± 4534.7^a,b^		
	Evening	2654.3 ± 3055.4	1922.2 ± 2821.7	6317.3 ± 4215.1^a,b^		
Glucagon (pg/mL)	Morning	121.7 ± 71.2	129.2 ± 63.6	142.1 ± 49.1^a^	1.35	0.2539
	Afternoon	111.2 ± 64.6	132.0 ± 82.2	142.0 ± 65.2^a^		
	Evening	135.2 ± 65.4	123.1 ± 83.8	140.8 ± 79.0^a^		

Mean ± standard deviation at each time-point. F and *p*-values refer to the condition by time interaction for each hormone.

^a^Indicates a significant difference from baseline values at a given time point.

^b^Indicates a significant difference from end of fast values at a given time point.

### 3.4 Beta-hydroxybutyrate

**FIGURE 5:**
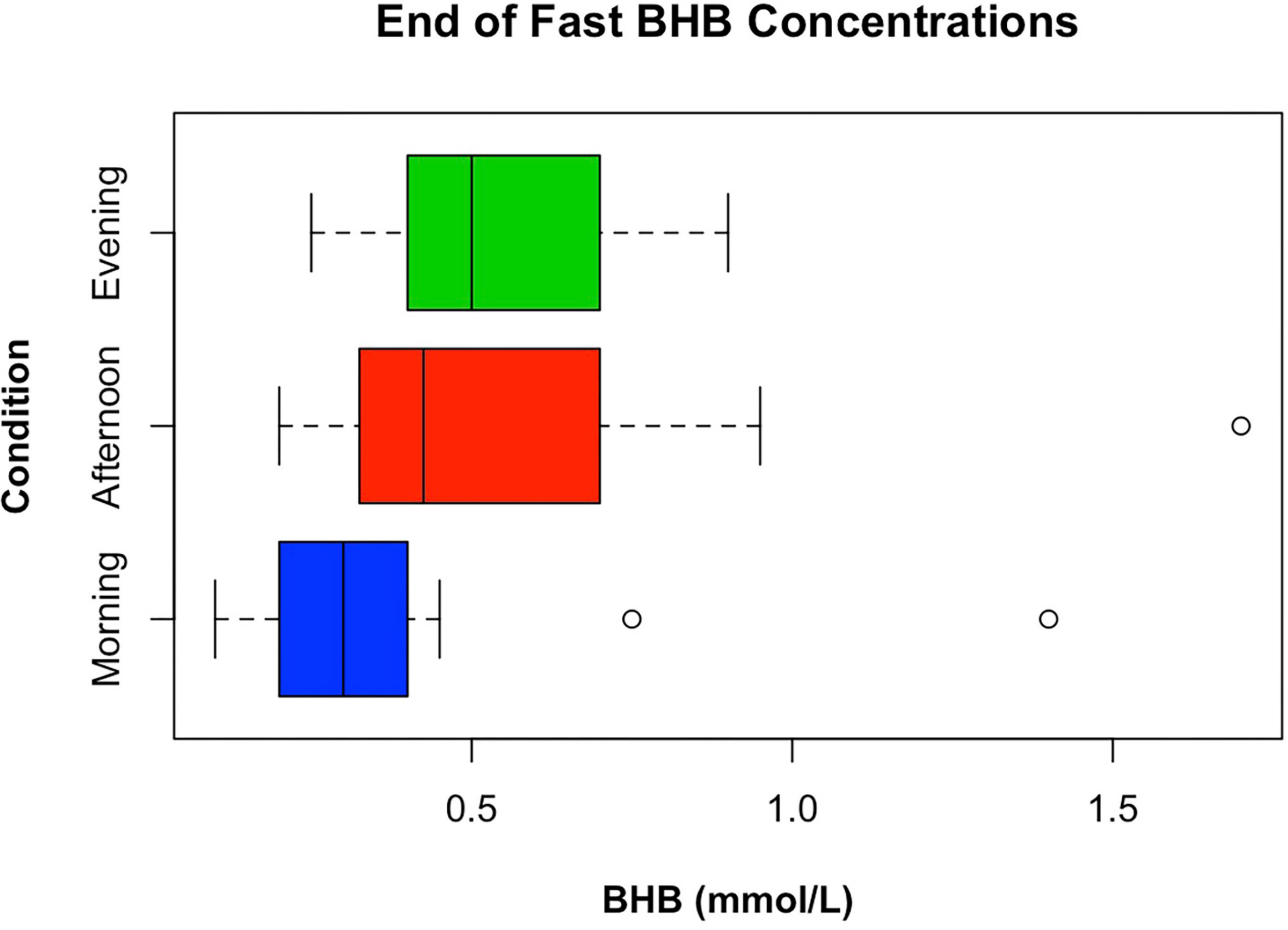
Box plot displaying differences in ketone concentrations between conditions at the conclusion of each fast to visually represent inter-individual variability in BHB concentrations.

A main effect of condition was found for BHB at the end of fasting [*F*(2, 46) = 4.48, *p* = 0.0166]. BHB was lower after the morning-started fast (0.36 ± 0.26 mmol/L) than after the afternoon [0.54 ± 0.32 mmol/L; t(46) = 2.67, *p* = 0.0104] or evening fasts [0.52 ± 0.20 mmol/L; t(46) = 2.51, *p* = 0.0156], with no difference between the afternoon and evening fasts [t(46) = 0.18, *p* = 0.8561]. BHB concentrations also showed inter-individual variability (see Figure 5). Thirteen participants had a BHB greater than or equal to 0.5 mmol/L at the conclusion of the evening fast, ten at the conclusion of the afternoon fast, and two at the conclusion of the morning fast.

## 4 Discussion

The timing of a 24-h fast influences glucose responses to a standardized meal, glucose regulation during the fast, and glucose tolerance upon refeeding. Our study found that initiating a fast in the afternoon optimized glucose regulation after the initial meal and over the subsequent 24-h period, while initiating a fast in the morning enhanced glucose tolerance post-fast compared to the evening fast. In contrast, the evening yielded the least favorable glycemic outcomes.

Previous studies have reported lower glycemic responses to an OGTT in the morning compared to the evening (12, 36, 37), though Takahashi et al. found similar glucose AUC responses to a mixed meal during both periods (38). Consistent with Takahashi et al., our study observed similar 2-h glucose responses to a standardized meal in the morning and evening. Unexpectedly, the afternoon fast resulted in lower glucose compared to either other condition. Elevated cortisol, growth hormone, or adrenergic hormones in the morning may have contributed to the greater rise in post-prandial glucose seen in the morning compared to the afternoon (39), while a progressive decline in insulin sensitivity throughout the day may explain elevated post-prandial glucose in the evening (36, 40–42). Nevertheless, these factors do not account for the lower glucose response observed in the afternoon. A potential mechanism to explain this observation is the second meal effect where morning food intake attenuates the glycemic response to a subsequent meal in the afternoon (43, 44). This phenomenon may counteract any potential reduction in insulin sensitivity to result in lower post-prandial glucose levels when a fast is initiated in the afternoon.

The lower glucose 2 h after the fasting-initiating meal corresponded to better glucose regulation throughout the 24-h fast. Mean glucose levels were significantly lower during the afternoon fast compared to the morning and evening fasts, which did not differ. When comparing fasting glucose in the morning, Triosi et al. reported lower fasting glucose in the afternoon and Saad et al. found lower glucose in the evening (45, 46). However, both these studies examined shorter fasting periods compared to the present study. To our knowledge, this study is the first to demonstrate lower mean 24-h glucose when a 24-h fast is initiated in the afternoon. Although glucose remained within the normoglycemic range across all conditions, even small reductions in fasting glucose may reduce cardiovascular risk, which increases linearly across normoglycemic levels (47).

Glucose variations during the nighttime contributed to a mean difference in 24-h glucose levels. As expected, nighttime glucose was higher in the evening versus the afternoon fast. Melatonin follows a diurnal secretion pattern, and the interaction between higher melatonin and post-prandial glucose excursions results in impaired glucose tolerance (15). Consequently, previous research has found that eating closer to bedtime can cause elevated glucose throughout the night (48–50), which is associated with greater risk of metabolic disorders (51). In the present study, nighttime glucose was likewise higher in the evening versus the morning fast; however, nighttime glucose was also higher in the morning versus the afternoon fast, which was unexpected. One plausible explanation for this finding is that the fasting duration before nighttime was longest when initiating a fast in the morning. Hepatic glycogen stores begin to deplete around 12 h of fasting (52), potentially triggering the release of counterregulatory hormones such as growth hormone to stimulate hepatic gluconeogenesis (53). Glycogen depletion coinciding with the natural nocturnal elevation in growth hormone may have augmented this hormonal response to raise blood glucose (54).

In contrast to glucose regulation while fasting, glucose tolerance upon refeeding was most favorable after the morning fast, aligning with our hypothesis. We observed that the glucose AUC was lower in the morning fast than the evening fast. In contrast, the afternoon fast did not differ from the other two conditions. These results support other research indicating lower glucose AUC in the morning than the evening after an OGTT (11, 35) but differ from most studies that observed different responses between the morning and afternoon (13, 37, 46). However, our study is the first to conduct OGTTs after 24 h of fasting at various times of day, thereby eliminating the impact of any previous food consumption. Prolonged fasting appears to maintain the diurnal variation of glucose tolerance between the morning and evening, with similar glucose tolerance to both conditions in the afternoon, indicating that glucose tolerance upon refeeding may be optimized when initiating a 24-h fast in the morning.

Prolonged fasting induces a metabolic shift from carbohydrate oxidation to fatty acid and ketone body utilization, with BHB serving as a marker of ketogenesis (52). Ketosis (defined as ≥0.5 mmol/L) (55, 56) is associated with several health benefits, including improved mitochondrial function (57), reduced oxidative stress and inflammation (58), and enhanced neuroprotection and longevity (57). Our study identified a diurnal variation in BHB concentrations following 24-h fasts initiated at different times of day. Despite fasting for 24-h in each condition, participants were not, on average, in ketosis at the conclusion of the morning fast. In contrast, BHB concentrations on average had similarly exceeded the ketosis threshold at the end of both the afternoon and evening fasts. These findings suggest that initiating a 24-h fast in the afternoon or evening may more effectively promote the metabolic switch to ketogenesis, potentially enhancing the physiological benefits associated with ketosis.

This observation aligns with other findings in the literature on diurnal ketogenesis rhythms (59). These results suggest that the highest concentrations of BHB are observed in the evening (60), reaching a peak around midnight (61, 62). These results also show a steady decline in BHB from 2 am until 8 am, reflecting reduced ketogenesis as the night progresses (63). These changes in ketone production may be influenced by fluctuations in blood glucose levels, as the two variables are inversely related (64). Glucose appears to be highest at the conclusion of the morning fast which is likely driven by the early morning rise in glucose that may result from elevated cortisol, growth hormone, or adrenergic hormones during this time (39), which may in turn reduce BHB production at that time.

Insulin plays a key role in regulating blood glucose and BHB production. Under normal physiological conditions, insulin follows a diurnal rhythm characterized by a morning peak and a gradual decline to a trough in the evening, even while fasting (65). However, this pattern can be blunted by prolonged fasting (46, 65, 66), which can also transiently delay insulin secretion following an OGTT (66, 67). In addition, obesity has also been shown to disrupt typical insulin variations and alter responses to an OGTT at different times of day (68–70). These factors may have contributed to the absence of the expected insulin rhythmicity observed in this study. Moreover, obesity is also associated with an attenuated reduction of insulin while fasting (71–73), which may explain the stable insulin levels seen across each fast. Contrary to our hypothesis, insulin concentrations did not differ between fasting conditions at any point during the fast or refeeding periods. This finding suggests that observed variations in glucose regulation and glucose tolerance may be explained by changes in insulin sensitivity rather than circulating insulin levels.

Glucagon also plays a role in regulating glycemic control and fat oxidation. Similar to insulin, no diurnal variations in glucagon were observed in this study. Previous studies have shown diurnal changes in glucagon, but the research is mixed and limited (46, 74). There is currently no established circadian rhythm for glucagon (74), and the results of this study do not support diurnal differences in a fasted state. Additionally, glucagon remained stable over the course of each 24-h fast, which was anticipated (75–78); however, there were also no changes in glucagon following the OGTT. Glucagon suppression is generally expected to occur after an OGTT (79), but obesity appears to blunt this suppression (80). Ultimately, the timing of a 24-h fast followed by refeeding does not appear to influence glucagon dynamics.

## 5 Strengths and limitations

This study provides valuable insights into the metabolic responses to a 24-h fast; however, several limitations warrant consideration. The free-living design introduced variability in participant behavior, as physical activity, sleep duration, and day of the week for fasting were not strictly controlled or objectively verified. Although participants were instructed to maintain typical behavior during each fast, these uncontrolled factors may have contributed to inconsistent results due to inherent differences in daily routines. Additionally, while participants were required to fast for at least 4 h before laboratory visits, participants fasted overnight prior to the morning fast, and the macronutrient composition and portion size of the meals prior to any fast were not standardized. In female participants, menstrual cycle phase was not controlled, potentially introducing further variability. Despite these limitations, the naturalistic setting enhances the ecological validity and generalizability of the findings. The randomized crossover design employed in this study mitigates some of these confounding factors, strengthening the reliability of the results.

Furthermore, only three blood draws and one BHB measurement were performed per fasting condition, limiting our ability to capture dynamic insulin, glucagon and BHB fluctuations during fasting and refeeding. More frequent sampling could have provided greater insight into post-prandial and diurnal variations in insulin, glucagon and BHB levels but was deemed impractical in this free-living study, as additional measurements would have disrupted participants’ sleep or daily activities. Finally, the OGTT results should be interpreted with caution due to missing CGM data for 18 of 73 fasting conditions, due to CGM removal before complete data consolidation. The mixed-effects model used to analyze glucose AUC accounted for these missing data under the missing at random (MAR) assumption via maximum likelihood estimation. However, the missing data may have reduced statistical power, potentially underestimating differences in glucose tolerance. Despite this, our results align with prior studies on glucose tolerance, supporting their validity. Future studies with more frequent sampling and robust glucose monitoring protocols would better characterize the dynamic metabolic responses to fasting and refeeding following prolonged fasts.

Despite the limited assessment of postprandial responses, this study has several key strengths. The use of a randomized crossover design allows each participant to serve as their own control, which minimizes inter-individual variability and enhances statistical power. Additionally, most circadian research on hormones related to glycemic control focuses on postprandial fluctuations, whereas this study is among the first to eliminate the confounding effects of food intake on hormone concentrations and the first to examine circadian variations at the conclusion of a 24-h fast. Furthermore, the use of CGMs provided glucose measurements every 15 min, offering detailed insights into glucose variations over the entire fasting period.

## 6 Conclusion

This study underscores the role of circadian timing in fast-based interventions as glycemic responses to a 24-h fast in individuals who have overweight or obesity vary based on the time of day that fasting is initiated. Among the three fasting conditions, the afternoon was associated with the most metabolically advantageous combination of glycemic outcomes and metabolic switching while fasting. Although glucose tolerance upon refeeding was most optimal after the morning compared to the evening, it was similar to glucose tolerance after the afternoon. In contrast, while the evening fast supported metabolic switching, it was associated with the least favorable combination of glycemic outcomes. These glycemic patterns across fasting conditions appear to occur independently of circulating insulin and glucagon concentrations.

Aligning fasting periods with circadian fluctuations may enhance the metabolic benefits of prolonged fasting. For individuals practicing alternate day fasting, beginning a fast in the afternoon may provide the greatest metabolic benefits and potentially reduce the cardiovascular risk associated with even modest elevations in fasting blood glucose. While direct extrapolation of these results to time-restricted eating practices is limited due to the extended fasting window in this study, initiating a time-restricted eating window in the afternoon rather than the evening may similarly improve glycemic outcomes throughout the remainder of the day.

The findings of this study emphasize the need for additional research into hormonal dynamics while fasting. Understanding how fasting affects circadian rhythmicity and the persistence of these effects would provide greater insights for optimizing feeding and fasting windows. Additionally, more research is warranted on the time course to ketosis when initiating a 24-h fast at different times of day, as ketosis onset and duration remains unclear during each fasting condition. A more precise characterization of these dynamics could inform more effective fasting strategies to further maximize the metabolic benefits of prolonged fasting.

## Data Availability

The datasets analyzed for this study can be found in the OpenScience Framework (doi: 10.17605/OSF.IO/TD2YU).
